# Suicidality and Self-Harming Behaviors in Patients with Prader-Willi Syndrome (PWS): Case Report and Literature Review

**DOI:** 10.1155/2021/2527261

**Published:** 2021-10-11

**Authors:** Val Bellman, Zargham Abbass, Ramsa Sohail, Syed Jafri

**Affiliations:** ^1^Department of Psychiatry, University of Missouri-Kansas City, USA; ^2^Shaikh Zayed Medical and Dental College, Lahore, Pakistan; ^3^University of Illinois Urbana-Champaign, USA

## Abstract

Prader-Willi syndrome (PWS) is a rare neurodevelopmental disorder which is often associated with significant behavioral challenges and poor intellectual functioning. Research has shown that individuals with PWS are more likely to experience mental health problems, have higher relapse rates, and are at risk of self-harming behavior. Although PWS is associated with mild intellectual disability, which in itself confers a higher mortality rate, suicidality in this population is so far unreported in the literature. We present the case of an 18-year-old male patient who was admitted to our facility following exogenous insulin administration with suicidal intent. The main clinical characteristics, self-harming behaviors, and suicide risk factors of patients with PWS are discussed in this report. The article's objective is to redirect clinicians' attention to carefully screen and treat the underlying behavioral problems in PWS patients.

## 1. Introduction

Prader-Willi syndrome (PWS) has been a subject of interest for many years, largely due to the unusual insatiable appetite common amongst those who are affected. In the United States, epidemiological records indicate the sporadic nature of the disease. Brito et al. [1] record a prevalence rate of 1 in 16,000, while Butler et al. [2] reported a prevalence of 1 in 24,590. Generally, prevalence estimates fall from 1 in 10,000 to 20,000 individuals in the United States, while global incidence rates range from 1 in 10,000 to 1 in 30,000 [3]. Bohonowych et al. [4] found no contrasting differences in gender and ethnicity. However, they further propose that Black individuals' rate of growth and facial characteristics are less affected than those of White people. These differences are insignificant given that the mortality rate is the same across all ethnicities [4].

Obesity complications such as sleep apnea and type II diabetes are major contributors to the mortality rate of people with PWS. Brito et al. [1] suspected a lower heart rate in slow-wave sleep than normal individuals due to modulation by a parasympathetic pathway triggered by a failure in cardiac autonomic balance. Differences in sexes are absent as PWS is a result of chromosomal 15's proximal arm losing paternal copy in domain 15p11-13.

The somatic component of PWS is only one aspect of this disorder, and there is vast literature on the psychological and mental health components of PWS. In a 2017 study, Butler et al. [5] analyzed causes of death amongst PWS patients. Out of 312 cases in which a precise cause of death was identified, none were due to suicide. The most common cause was respiratory failure followed by heart disease and GI-related problems. This limited study might give the impression that those affected by PWS are not at risk of suicide; however, several other studies point to the contrary. A 2012 study conducted by Skokauskas et al. [6] sought to analyze the mental health of children presenting with PWS. The study showed that children with PWS had more severe somatic, affective, social, and thought problems and were more withdrawn/depressed in comparison to controls. Children and adolescents with PWS also exhibited more comorbid psychological problems than their peers matched for age, IQ, and gender. It is well documented that those with affective disorders and those suffering from somatic symptoms are at higher risk of suicide and suicidal ideation [7, 8]. Given that PWS patients often present with such symptoms and disorders, there is a strong possibility that PWS is likely a risk factor for suicide.

## 2. Case Presentation

The patient is an 18-year-old African American male with a documented history of Prader-Willi syndrome with mild intellectual disability and type I diabetes mellitus (DM) who presented to the emergency department (ED) multiple times over the course of two months after attempting suicide via multiple methods.

### 2.1. First Encounter

The patient went shopping with his mother and became upset that he was not getting a high carbohydrate meal. He then became behaviorally dysregulated later on and refused to step out of the car. Later that evening, he refused to take his insulin. He was then found by his mother at nighttime somnolent and reported overdosing on his home insulin. This event led to him being brought to the hospital. Psychiatry was consulted to assess for continuous suicidal thoughts and guide disposition. The mother ended up enacting the durable power of attorney (DPOA) for the patient. The patient refused to take any psychotropic medications, and the team recommended that the patient would not benefit from acute inpatient psychiatric admission at this time; the patient was then discharged.

### 2.2. Second Encounter

The patient's mother brought him to the ED for an assessment of violent aggressive behavior. The patient denied any aggressive episodes and was not able to recall any. He did admit to having a displacement reaction in which he used to hit his pillow when he got upset.

According to the mother, the patient was living at a homeless shelter. Prior to that, he was living with his sister until she killed herself, and he was not able to live in that house as he kept experiencing flashbacks. The team once again did not see the benefit of admitting him to an acute inpatient psychiatric unit as he was not at risk of harming himself or others at the time of assessment. He was discharged back to the homeless shelter.

### 2.3. Third Encounter

The patient was admitted to the ED, and psychiatry was consulted as the patient attempted to wrap a laptop cord around his neck and attempted to jump out of a moving vehicle. He was agitated in the ED requiring as-needed meds for agitation (i.e., haloperidol and lorazepam).

The patient told his treatment team that his mother triggered this episode but was unable to elaborate on it further. Due to this suicide attempt and continuous suicidal ideation, the patient was admitted to the acute inpatient psychiatric hospitalization involuntarily for safety.

He was started on aripiprazole 5 mg orally and was placed on 1 : 1 observation due to being a potential victim risk. He was given a diagnosis of unspecified depressive disorder, as he had been experiencing some depressive symptoms since age 12. During his stay at the inpatient unit, he did not endorse perceptual disturbances, depression, and anxiety, nor did he exhibit agitated behavior. He was then discharged to the homeless shelter.

### 2.4. Mental Status Examination

The patient appeared his stated age, mildly obese with apparent gynecomastia. He exhibited intermittent eye contact. His speech was normal during conversation but was childish in tone with noticeable changes in pitch between phone call and conversation.

### 2.5. Past Psychiatric History

The patient has a history of multiple inpatient psychiatric hospitalizations in different facilities in the area. No formal diagnosis of depression, primary thought disorder, or bipolar disorder was given in the past. His primary symptoms have always been in relation to his behavioral dysregulation and compulsive/impulsive eating.

He was diagnosed with Prader-Willi syndrome at the age of 13 years. He did not receive therapeutic treatment (e.g., growth hormone); had sleep difficulties, hyperphagia, and developmental delays (as per the mother), and lack secondary characteristics.

There was no outpatient psychiatric follow-up. The patient refused to take psychotropic medications; however, he has tried guanfacine, hydroxyzine, and fluoxetine. The length of these trials is unknown. Fluoxetine exacerbated his suicidal ideations. He was prescribed trazodone for sleep. According to the chart review, he has been on escitalopram (two trials with unknown outcomes) as well. He has no substance abuse history.

### 2.6. Social History

He did poorly in school, requiring an individualized education plan (IEP) for graduation. There were reports of behavioral disturbances related to being bullied in high school. He has been unable to hold a job and lives with his mother who is also his support.

### 2.7. Discharge

During the past three admissions to the ED, the patient was admitted to the inpatient psychiatric hospitalization only once. There was an absence of follow-up in the outpatient setting after his discharge.

## 3. Discussion

### 3.1. PWS: Overview

PWS is a complex multisystem genetic disorder manifesting with behavioral, physical, and learning deficits. Although exhibiting heterogeneity, PWS was the first microdeletion condition to be identified on chromosomal analysis with high resolution. In addition, it is a uniparental-disomic and gene-imprinting disorder epitomized by distinct phenotypic behavior [1]. The major signs consist of infantile hypotonia, intellectual disability, hypogonadism, and short stature. The individual may also have signs and symptoms of obesity and various endocrine abnormalities [9].

Cassidy and Geer [10] assert that neonate behavior in regard to hypotonia is invariably correlated with reduced sucking ability. Reduced or absent infantile reflexes at birth also contribute to lethargic tendencies. Furthermore, the characteristic hypotonia improves over time with the infant sitting and standing at one and two years old, respectively [4]. Depending on the level of injury, the presentation of hypotonia extends into adulthood in the form of diminished bulk and tone of the muscle.

PWS symptoms of developmental delay are present in 90 to 100 percent of infants [10]. They have difficulty learning resulting from impaired intellectual abilities that are below normal with an IQ below 70. Although the majority of patients have apparent articulation difficulties, they do have strength in expressive language (verbal) that gradually improves with age.

**Figure 1:**
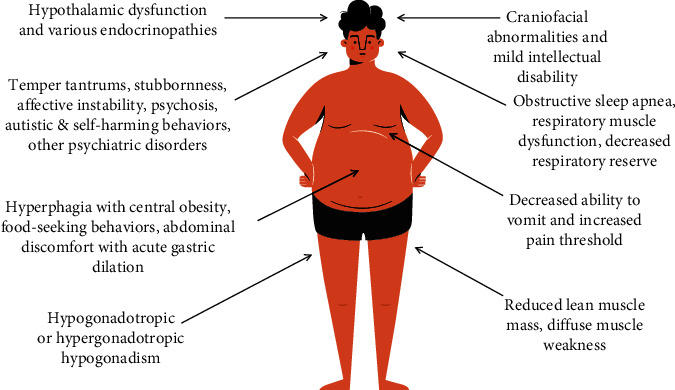
Clinical features of Prader-Willi syndrome (PWS).

Bray et al. [11] concluded that cognitive impairment and central obesity become more prominent later. Specifically, increased body mass index is triggered by hyperphagia in the one- to six-year-old age range. Hyperphagia also leads to marked food-seeking behavior such as eating garbage and stealing money to buy food. In the absence of growth hormone supplementation, most patients present with stunted pubertal height. In untreated individuals, average heights are 155 cm and 148 cm for males and females, respectively [2]. Hypothalamic dysfunction and various endocrinopathies are implicated in numerous manifestations of this syndrome, including hyperphagia, hypogonadism [10], temperature variability, obstructive sleep apnea, and insulin regulation of blood sugar [4].  Figure 1 summarizes the main clinical characteristics of patients with PWS.

### 3.2. Mortality in Prader-Willi Syndrome

Individuals with PWS carry a risk of significant morbidity and mortality compared to the general population. A study carried out by Whittington and colleagues [3] in one UK health region calculated an approximate death rate of 3 percent per year for those with PWS compared to 1 percent per year for the general population. The mean age of reported deaths in PWS is 29.5 years; 20 percent of deaths occur below the age of 18 years, and mortality is often unexpected [3].

In 2017, a publication by Butler and colleagues [5] extensively reviewed data from 1973 to 2015, with a priority of describing the causes of death more specifically. Four hundred and eighty-six families reported death to the Prader-Willi Syndrome Association (PWSA). These individuals were between the ages of 2 months and 67 years. Seventy percent of these deaths occurred in adulthood. Respiratory failure was the most common cause of death (31 percent) in PWS in both adults and children. Cardiac problems were the second leading cause at 16 percent, primarily in adults, due to obesity-related heart failure rather than due to coronary disease. These are not surprising findings when uncontrolled weight has long been the greatest challenge in this syndrome. A surprising 10 percent of deaths were due to GI problems, and GI-related death occurred at all ages. Choking was more common in males, in part due to rapid eating when sneaking food but also complicated by the lack of saliva and the ineffective swallowing that is often part of the syndrome [5].

The causes of death reported in studies are different in adults and children with PWS. A study conducted in 2004 by Schrander-Stumpel et al. [12] on 27 individuals who had PWS and subsequently died noted that respiratory distress and sudden death associated with dysregulation of temperature were more common in infants and children whereas deaths in adults with PWS were due to obesity and its complications, including cardiovascular problems, sleep apnea, diabetes mellitus, and hypertension. Gastric dilation was also observed in adults, but not in teenagers [12].

There are reports of sudden death in children with PWS who were started on growth hormone therapy [13]. The causes of death in this subset were attributed to worsening of sleep disturbance. Moreover, Lamb and Johnson [14] reported premature development of atherosclerosis with severe coronary artery disease in a patient aged 26 years with Prader-Willi syndrome, morbid obesity, and non-insulin-dependent diabetes mellitus.

In summary, causes of death seen in infancy or young childhood in PWS are more likely to be related to respiratory failure, aspiration, infection, hyperthermia due to dysregulation of temperature control, and choking rather than obesity-related factors, while cardiac disease and failure, pulmonary thromboembolism, accidents, sepsis, and obesity-related complications are more commonly found in adolescents and adults. Males may be more likely to engage in aggressive or risky food-seeking behaviors than females, particularly in childhood, while females may be more likely to suffer obesity-related morbidity.

### 3.3. Self-Harming Tendencies in PWS

It has long been thought that PWS-related intellectual disability could act as a shield against suicidality due to the assumed inability of those with intellectual disability to conceptualize, plan, and carry out suicide. However, the studies that have been done on this topic have contradicted this idea, finding that those with and without intellectual disability are subject to the same risk factors for suicide and that the signs of suicidality are similar [15, 16]. Furthermore, individuals with intellectual disability are more likely to be exposed to the risk factors for suicide. Additionally, those with intellectual disability are more likely to experience childhood abuse and social ostracization, contributing to mental illness and suicidal ideation [16].

This demonstrates that intellectually disabled individuals respond to the same triggers of suicidality as the broader population but are more at risk of suicidal behavior due to their higher exposure to these triggers. A 1988 study of 150 admissions of handicapped children to a psychiatric hospital found that nearly 40 percent of the sample experienced past and/or current maltreatment, including physical abuse, neglect, and sexual abuse [17]. In a study of 233 children and adolescents with intellectual disability, researchers found that 20 percent had a history of suicidal ideation [18]. Mildly intellectually disabled individuals were found to be at a much higher risk for anxiety and suicidality when compared to those with moderate or severe intellectual disability [16].

However, the difficulty in assessing suicidality lies in the fact that it is often difficult for those with intellectual disability to articulate their thoughts and feelings clearly or communicate effectively with their caregivers. In fact, a study of suicidal behavior in adults with intellectual disability found that 70 percent of the patients were not thought to be suicidal by their caregivers or relatives [19]. As these individuals are generally considered the most reliable source of data, this presents a problem in recognizing the need for therapy and professional help for suicidal individuals with intellectual disability.

Instead, warning signs for psychological distress, especially in children and adolescents, present as maladaptive behaviors rather than verbalized statements and may go unrecognized. These include self-injury (81 percent of 62 participants according to Symons et al. [20]) in the form of skin picking, mostly on the legs and head, sleep disorders, insistence on routine, ritualistic and compulsive behaviors [21], and frequent temper tantrums. Skin picking was more severe and involved more body parts in individuals with the 15q11-q13 deletion than in those with maternal uniparental disomy subtype [20]. Moreover, this behavior was observed to happen when patients were alone, suggesting that this behavior may be sensitive to environmental and parental influences. The data indicated that itch and pain processes appeared to underlie skin-picking behaviors in PWS, suggesting that interoceptive disturbance may contribute to the severity and maintenance of abnormal skin-picking behaviors in PWS [22]. It was concluded that skin picking was more common in individuals with PWS than in those with ASD or intellectual disabilities [23]. Head banging was the same in all three groups, and over time, those with PWS showed an increase in behaviors which included hitting and/or biting themselves [23].

These behaviors extend well into adult life and cause significant social and behavioral challenges for these patients, including but not limited to social withdrawal, feelings of isolation, and bullying by peers [24]. Of all these factors, bullying and not being able to be at par with others seem to be significant contributing factors to feelings of sadness, inadequateness, and suicidality amongst people with PWS.

In a study conducted by Skokauskas et al. in 2012 [6], children with PWS were demonstrated to have more severe somatic, social, and thought problems and were more withdrawn/depressed in comparison to controls. Other authors suggested that people with PWS have difficulty with core, receptive, and expressive language skills, interpreting emotional valence in faces, playing with children of their own age, and understanding personal space and a developmental delay in the theory of mind [25, 26]. Moreover, Einfeld et al. showed that patients with PWS were more likely to be antisocial and behaviorally disturbed [27].

In 2006, a study by Soni et al. [28] studied 119 cases of PWS of which 46 suffered from a psychiatric illness. The study concluded that individuals with maternal uniparental disomy (mUPD) had a higher rate of psychiatric illness than those with paternal deletion. In both genetic subtypes, the psychiatric illness resembled an atypical affective disorder with or without psychotic symptoms. Those with paternal deletion were more likely to have developed a nonpsychotic depressive illness, and those with mUPD developed a bipolar disorder with psychotic symptoms. Individuals with the paternal deletion subtype and psychotic illness had an increased family history of affective disorder. This was confined exclusively to their mothers. Individuals with mUPD had a more severe course, increased risk of recurrence and incidence, more episodes, and a poor response to medication. Selective serotonin reuptake inhibitors (SSRIs) provided benefit to the patients; mood stabilizers, however, proved to not be beneficial in this population [28].

The lack of standardized guidelines for suicide risk assessment of individuals with intellectual disability compounds this problem. Current screening instruments designed for children and adolescents without intellectual disability have limited utility for the population with disability. This is due to the complexity of these instruments in terms of reading comprehension, complex response formats, and the need for abstract thinking [19].

It has been clearly proven that individuals with PWS suffer from various psychiatric illnesses including anxiety, depression, psychosis, aggression, and compulsiveness. Current guidelines of treatment focus on identifying the nature of the illness and treating it appropriately with antipsychotics, antidepressants, mood stabilizers, anticonvulsants, beta blockers, and alpha 2 agonists. Yaryura-Tobias et al. [29] showed in four PWS patients with hyperphagia and self-injurious behavior that SSRIs and phenothiazines improved self-injurious behavior (SIB) symptoms but were ineffective in controlling appetite satiation. Research done by Shapira et al. [30] reported attenuation of SIB in three PWS adults treated with topiramate in an 8-week open-label trial. Currently, intranasal oxytocin is being studied for individuals with PWS as studies have shown its use to be associated with a reduction in appetite drive and improvements in socialization, anxiety, and repetitive behaviors [31]. However, long-term studies with a greater number of participants are required to validate existing research and establish a framework to guide clinical practice. Additional research is required to evaluate how medical treatment can be used in concert with behavioral therapies to treat the specific behavioral patterns like self-injury seen in individuals with PWS. A better understanding of the biological variables underlying the occurrence and maintenance of such behavior is also needed.

## 4. Conclusion

Patients with PWS frequently engage in numerous forms of self-harming behavior, which can be seriously dangerous—physically and emotionally. Despite this, there continue to be challenges in assessing suicide risk in the population of intellectually disabled individuals and a lack of standardized screening and diagnosis procedures. Moreover, it appears that suicide rates in this population are still significantly underestimated. Risk factors (e.g., history of prior psychiatric hospitalizations, comorbid affective disorders, and physical disabilities) are associated with suicidal behavior in these patients. Because of this, clinicians and other healthcare professionals should be aware of the risk factors for suicide and signs of suicidal behavior and be able to intervene when necessary.

Unfortunately, there is limited evidence to guide prevention and behavior modification strategies for suicidality in patients with PWS; however, our observations suggest that the borderline level of intellectual functioning and lack of appropriate supervision may contribute to the severity of prosuicidal behaviors in PWS.

## Data Availability

Data sharing is not applicable—no new data is generated.
